# Adjustment of self-initiated and organizational expatriates: The moderating role of cross-cultural training

**DOI:** 10.3389/fpsyg.2022.1044040

**Published:** 2023-01-10

**Authors:** Muhammad Noman, Muhammad Safdar Sial, Sarminah Samad, Rita Yi Man Li, Miao Shi

**Affiliations:** ^1^School of Journalism and Communication, Wuhan University, Wuhan, China; ^2^Department of Management Sciences, COMSATS University Islamabad (CUI), Islamabad, Pakistan; ^3^Department of Business Administration, College of Business and Administration, Princess Nourah Bint Abdulrahman University, Riyadh, Saudi Arabia; ^4^Sustainable Real Estate Research Center, Department of Economics and Finance, Hong Kong Shue Yan University, Hong Kong, Hong Kong SAR, China; ^5^School of Management, Pass College of Chongqing Technology and Business University, Chongqing, China

**Keywords:** cross-cultural adjustment, self-initiated expatriates, organizational expatriates, cross-cultural training, Pakistan

## Abstract

Globalization and the international labor movement made the ability to work anywhere globally. These individuals are usually organizational expatriates (OEs) deployed to overseas assignments by their employers or self-initiated expatriates (SIEs) who choose to relocate and work in a foreign country. Therefore, this study examines and contrasts the variations in cross-cultural adjustment (CCA) between Chinese SIEs and OEs in Pakistan. Furthermore, it aims to understand how cross-cultural training (CCT) influences the adaptation of Chinese SIEs and OEs. Data were obtained from 35 Chinese expatriates with 17 SIEs and 18 OEs employing a qualitative technique and were analyzed *via* thematic analysis in MAXQDA 20. According to the study, both SIEs and OEs face distinct hurdles to their CCA in Pakistan, including cultural taboos, language obstacles, and work variations. While numerous aspects that will favorably affect their CCA, including the accessibility of necessities, the friendliness of the Pakistani people, and the brethren relations between Pakistan and China, assist them in making a smooth transition to life in Pakistan. Furthermore, the results indicate that the mediating role of pre-departure CCT and the host country mentors’ support and facilitations acquired through online resources contribute to both Chinese SIEs and OEs’ CCA in Pakistan.

## 1. Introduction

Globalization has ushered social, economic, cultural, technological, and educational variations (Altman and Shortland, 2008). Consequently, many global corporations can now establish operations overseas to compete in the international economy (Srivastava and Pandey, 2012). In recent years, China has increased its involvement in several nations worldwide (Buckley et al., 2018; Jackson and Horwitz, 2018). As a result, many Chinese expatriates have relocated worldwide (Zhang and Fan, 2014; Cooke et al., 2017). China launched several significant international investments, including the Belt and Road Initiative (BRI; Huang, 2016), in 2013. The span of this venture extends from China through South East Asia, East Africa, West Asia, Greece, Venice, and finally, Rotterdam (Ahmad, 2016), emphasizing spatial connection and economic growth. In recent years, the mobility of more than 30 million Chinese expatriates throughout the globe (Textor, 2021) has sparked a greater interest in Chinese expatriates (Charoensukmongkol, 2021; Guang and Charoensukmongkol, 2022). Several studies have analyzed the difficulties faced by Chinese expatriates in Settings (Bader and Schuster, 2015; Abugre, 2018). As a component of the Belt and Road Initiative (BRI), the China-Pakistan Economic Corridor (CPEC; Syed, 2018; Bari et al., 2019) was recently launched, providing a rare chance to study Chinese expatriates’ adaptation to a hitherto unexplored context: Pakistan. Since the inception of the (BRI), many Chinese companies and expatriates have relocated to Pakistan, adapted to Pakistan’s work and non-work sectors (Zhang and Fan, 2014).

The extant research on expatriate CCA has concentrated chiefly on Western expatriates (Olsen and Martins, 2009; Harrison and Michailova, 2012; Makkonen, 2016). To have a thorough knowledge of expatriate CCA, it is essential to recognize that expatriates face several psychological, behavioral, and somatic obstacles while relocating to a new distant nation (Firth et al., 2014; Maertz et al., 2016). In addition, there are substantial disparities between Western and Asian environments (Chuang et al., 2015; Tahir, 2018), which uniquely impact expatriates’ adjustment process. Nevertheless, research on expatriate CCA has concentrated chiefly on Western expatriates (Olsen and Martins, 2009; Harrison and Michailova, 2012; Makkonen, 2016). Asian expatriate CCA obstacles have received little attention (Ang and Tan, 2016; Nadeem and Mumtaz, 2018; Noman et al., 2020). When someone relocates to another country to work for one of the firm branches, they are referred to as expatriates (Guo et al., 2016). Expatriates with appropriate employment assets enjoy the benefits of foreign professional mobility and experience a smoother transition to living and working in the host nation (Dimitrova et al., 2020). The two main categories used to describe expatriates by scholars are “self-initiated expatriates” (SIEs) and “organizational expatriates” (OEs; Jokinen et al., 2008). Independent movers are known as self-initiated expatriates (SIEs). Their ambition is to find employment in a foreign country, and they are taking the necessary steps to make that happen (Linder, 2018).

In contrast, organizational expatriates (OEs) are those transferred overseas by their employers (Linder, 2018). Many scholars concluded that SIEs move overseas for individual reasons such as self-improvement, whereas OEs go foreign mainly to achieve professional or organizational objectives (Suutari and Brewster, 2000; Peltokorpi, 2008). Consequently, firms rely on expatriates who can handle assignments in other countries to sustain and improve their competitiveness (Dowling and Welch, 2005). According to Koo Moon et al. (2012), effective expatriate CCA is one of the critical foundations of an organization’s success. Through CCT, expatriates must get adequate knowledge, comprehension, and awareness of the acceptable norms and behaviors of the host country to improve their abilities (Wang and Tran, 2012). It also helps expatriates tackle intercultural settings (Littrell and Salas, 2005). Further, CCT is a set of formal guidelines designed to help workers improve their social and professional interactions with coworkers from diverse cultural backgrounds (Brislin and Yoshida, 1994). It fosters an expatriate’s cross-cultural understanding, allowing them to effectively interact with others from distinct cultural environments (Wang and Tran, 2012). The effective CCT may enable expatriates to become acquainted with the host nation’s culture, legislation, rules, working atmosphere, and other aspects. Thus, it aids their cross-cultural adaptation while operating overseas.

The studies on OEs have emphasized intercultural adjustment and various employment aspects, including work effectiveness and engagement (Hechanova et al., 2003; Bhaskar-Shrinivas et al., 2005). On the contrary, existing studies on SIEs have focused on motivational and professional life elements of self-directed international encounters (Richardson and Mallon, 2005; Al Ariss and Syed, 2011; Selmer and Lauring, 2011a). SIE scholars have given less consideration to CCA and employment aspects (Selmer and Lauring, 2011b; Froese et al., 2012), despite offering crucial information about the possibly more extensive expatriate community than OEs (Myers and Pringle, 2005). It’s also worth noting that scholars have conducted few studies comparing SIEs and OEs. These studies demonstrate that individual and employment variables, CCA, and work engagement vary amongst SIEs and OEs (Doherty et al., 2011; Froese and Peltokorpi, 2011). Moreover, little is understood about why there are disparities in critical expatriate consequences such as CCA and work satisfaction between SIEs and OEs. This study looks into these discrepancies between SIEs and OEs, the underlying causes of variations in CCA, and work satisfaction between SIEs and OEs.

The research significantly advances two crucial areas of the literature. First, the research investigates the distinctions between SIEs and OEs and their CCA in Pakistan’s diverse cultural atmospheres. Furthermore, the study’s sample consists of Chinese OEs and SIEs, working in Pakistan. By investigating the adjustment issues faced by Asian expatriates in an Asian context, this sample contributes to the international business literature. Due to their growing global presence, Chinese expatriates’ adjustment difficulties are also valuable. Second, research indicates that one of the essential elements of an overseas task is the ability to adapt to different cultures. Therefore, CCT plays a crucial role. The main goals of CCT techniques are to reduce culture shock and increase awareness of cultural diversity among expatriate workers while also assisting them in comprehending the value of culture (Eschbach et al., 2001). However, there is no previous research on this topic in Pakistan. The present study to close this research gap and advance our understanding of this topic. The results of this study may aid Multi-national corporations in creating appropriate CCT programmes that assist expatriates and their families in adjusting to the distinctive socio-cultural atmosphere of Pakistan. It may also encourage other research investigations on this critical topic. This study aims to examine the elements that influence Chinese SIEs and OEs’ CCA and, particularly, to determine the differences between the SIEs and OEs’ CCA process in Pakistan. Further, the study is interested in finding the degree of CCT that will help facilitate the Chinese SIEs and OEs; for instance, the moderating role of CCT in SIEs and OEs’ CCA.

This paper has been four sections. The first section includes a brief review of the main literature, while the second section discusses the methodology, including details about the interviews with Chinese expatriates and how the data were analyzed. The third section is the study findings. The fourth section presents the discussion of the study results. The final section provides a conclusion, practical implications, future research directions, and limitations of the study.

## 2. Literature review

### 2.1. Defining the term “SIEs” and “OEs”

When individuals leave their national borders to live and work temporarily in another nation, this process is known as expatriation (Richardson and McKenna, 2006; Rose et al., 2010). According to Suutari et al. (2018), the word “ex-pat” originates from the Latin term “ex-Patria,” which means “away from one’s nation.” It describes someone leaving their country searching for better employment and living opportunities. So, an expatriate is a person who plans to live and work for a specific amount of time in a foreign nation or culture (Holopainen and Björkman, 2005).

There are two types of expatriates looking for work abroad: organizational expatriates (OEs) and self-initiated expatriates (SIEs; Howe-Walsh and Schyns, 2010). When a parent company sends a highly qualified employee to work in a foreign subsidiary for a set length of time (often three to 5 years), these workers are known as “OEs” (McKenna and Richardson, 2007). Additionally, SIEs migrate and seek employment in a foreign nation without assistance from native firms (Selmer and Lauring, 2010; Tharenou and Caulfield, 2010). OEs are offered training and skills before moving overseas, whereas SIEs make their own expatriation determinations (Howe-Walsh and Schyns, 2010).

Literature reveals that SIEs are more prevalent and well-liked than OEs and that the quantity of SIEs is significantly greater than that of OEs (Biemann and Andresen, 2010; Swim et al., 2011). That is, since SIEs are free to commence their journey overseas and are not obligated to serve in specific companies, unlike OEs. The latter return to their firm when the foreign mission is done. Results revealed that SIEs comprise 65% of the professional workforce (Doherty, 2013). Additionally, the research found that 50 to 70% of expatriates fall within the category of SIEs (Jokinen et al., 2008; Peltokorpi and Jintae Froese, 2009; Swim et al., 2011).

### 2.2. Cross-cultural adjustment of SIEs and OEs

The term “CCA” describes how well an expatriate has adjusted psychologically and socially to live in a new culture (Black, 1988; Black et al., 1991). Expatriates tend to become more at ease and start integrating with the foreign culture through a procedure that entails uncertainties, minimization and transformation (Black, 1988). Through copying and practicing culturally acceptable behaviors, expatriates might lessen fear and confusion. Expatriates who have successfully adjusted to their foreign culture are often receptive to the host society’s customs. They can build on the foundation established by their own cultures in terms of new habits, values, and laws (Church, 1982). On the other hand, maladjusted expatriates are incapable or unwilling to adapt and change their behavior to the foreign nation’s habits, customs, and standards. Expatriates may consider foreign cultures lower than their own and their own culture overly favorable if they had unpleasant experiences there.

Addressing the CCA of expatriates, there is a substantial corpus of literature (Bhaskar-Shrinivas et al., 2005; Takeuchi, 2010). Black and Stephens (1989) recognized the three significant aspects of CCA in the research on expatriate adjustment: general adjustment, interaction adjustment, and work adjustment. The way expatriates adapt to living abroad is referred to as general adjustment. The process of adjusting to new social contacts is known as interaction adjustment. Expatriates’ integration into the workplace is a crucial component of work adjustment. Previous research on expatriate adjustment has frequently used these three elements, which have already been proven valid (Bhaskar-Shrinivas et al., 2005).

While an extensive study was undertaken on the CCA of OEs, fewer are revealed about the CCA of SIEs. Generally, the emphasis of cross-cultural studies on expatriates has been on OEs delegated by the MNEs’ corporate headquarters to the overseas subsidiary. The General Agreement on Trade in Services (GATS), signed by countries, establishes the guidelines for the mobility of specialists across national borders and the reciprocal validation of credentials (WTO, 2019; Guo et al., 2021). According to research by Peltokorpi and Jintae Froese (2009), SIEs are more likely to exhibit higher general and interaction adjustment levels than OEs. Regarding work adjustment, Froese and Peltokorpi (2011) discovered that SIEs are less satisfied with their professions than OEs. They suggested that SIEs’ pre-existing social networks with locals and stronger desire would favorably impact interaction and general adjustment. This is problematic because they did not experimentally evaluate the antecedents of SIEs’ adjustment. They predicted that SIEs get less assistance at the workplace, which would contribute to lower work satisfaction.

Additionally, Richardson (2006) and Vance (2005) suggested that families and spouses could favor expatriates’ adjustment. In conclusion, there are significant differences among SIEs and OEs in terms of CCA, and these differences in precursors may also exist across SIEs and OEs. Therefore, this study explores the differences between the SIEs and OEs and their CCA process in Pakistan’s work and non-work environments.

### 2.3. Cross-cultural training

The CCT has a considerable effect on their work performance. Since the advent of globalization, there has been a rising demand for CCT. According to research by Hunt et al. (2015), multicultural employees and fiscal gains are directly correlated, demonstrating the significance of CCT and its productivity. In other words, a more diversified workforce leads to better financial results. With the help of CCT, expatriates may better locate and adapt to their host settings to perform at a higher standard. The goal of CCT is to train individuals of one culture how to interact successfully and work with individuals of another culture and to assist them in efficiently adjusting to their new place (Tahir, 2022). This training includes cultural orientation, language learning, social skill building, interactions, etc. (Krishnaveni and Arthi, 2015).

The study of expatriates’ adjustment has dominated the field of cross-cultural literature (Bruning et al., 2012; Abdul Malek et al., 2015; Kawai and Mohr, 2015; Selmer and Lauring, 2015; Zhang and Oczkowski, 2016). When performing their duties, expatriates encounter difficulties living and working in a foreign country (Black et al., 1991). The challenges in transitioning to these circumstances sparked a heated discussion about ways to increase efficiency in the host nation and reduce the failure rate of an expatriate mission. According to Black and Stephens (1989), expatriates are deemed acclimated to the host culture if they feel at ease, psychologically satisfied, and unbothered by any of the three adjustment components: general, interaction, and work adjustment.

The contribution of CCT professionals working abroad has been substantial. The researchers recognized that CCT plays a crucial role in expatriates’ successful international adaptation (Waxin and Panaccio, 2005). Additionally, CCT is frequently referred to as a strategy for enhancing expatriates’ ability to perform well overseas (Forster, 2000). According to Black and Mendenhall (1990), CCT has been implemented to improve interactions between people of different cultural backgrounds. Expatriates’ ability to adapt to their new surroundings and increase their understanding of local culture are both aided by training (Porter and Tansky, 1999). Caligiuri et al. (2005) stated that CCT was designed to assist individuals in feeling secure while living and working overseas, boost their acculturation, and improve their capacity to detect and comprehend diverse cultural contexts. Appropriate planning, like providing these expatriates with CCT before they depart, can help mitigate or eliminate many potential problems during their time abroad. The CCT strives to accomplish three primary goals. The first is that CCT ought to assist expatriates in anticipating (in ahead) appropriate methods of carrying out work and appropriate cultural behavior in the host nation. The second goal is to increase expatriates’ cognitive ability to deal with unexpected occurrences and disputes caused by unanticipated circumstances and acts. The third objective of CCT is for expatriates to have realistic assumptions concerning their living and working conditions in the host nation (Caligiuri et al., 2001). In addition, Mondy and Noe (2005) proposed that various types of pre-departure and CCTs focusing on the host nation’s language, culture, living conditions, and social norms will aid expatriates in adjusting to the host culture with ease. Therefore, this training aims to increase expatriates’ understanding of the host culture and familiarize them with cultural diversity.

#### 2.3.1. Pre-departure training and informal learning

The initial phase is pre-departure training, and experts discovered that pre-departure CCT is quite successful once expatriates reach overseas (Mendenhall, 1999). First, CCT equips expatriates with the essential information they need as soon as they arrive at their location, such as cultural etiquette, business etiquette, appropriate clothes for the environment, mandatory and optional traditions for individuals and solid language abilities (Avril and Magnini, 2007). Before leaving on a mission, expatriates should be informed that they may encounter uncertain situations. They may see these situations as instructional experiences. If they encounter circumstances where the outcome is obscurely successful, they should design a path for development rather than trying to escape (Avril and Magnini, 2007).

Informal learning is a constant process *via* which knowledge, skills, behaviors, and experiences are acquired. This might be intentional or unintentional, and it encourages the development of new ideas and methods. We have access to an infinite number of materials for informal learning since we live in a digital world. Informal learning is pursued *via* exposure to real-world experts and colleagues in the workplace and through online resources, mentorship programs, and other technical networks. In addition, the expatriates often organize those informal training approaches individually, while the employer may occasionally provide such training (Brewster and Pickard, 1994). Informal learning utilizes a variety of strategies and tools: people share occupational safety knowledge *via* Twitter (Song et al., 2022), construction practitioners and government officials share the construction safety knowledge *via* blog (Li and Poon, 2011) and Weibo (Zeng and Li, 2022) and residential safety *via* Twitter (Li et al., 2022). According to Bear (2008), the majority of expatriates utilize internet-based tools like different Social media websites, e.g., Google, Facebook, YouTube, Whatsapp, Wechat, and Twitter, as informal learning resources to understand the culture and the new country.

Additionally, informal learning is acquired *via* regular employment and social interactions. According to Sambrook (2005), informal learning occurs in the workplace *via* appraisal, practice, and rather than through participation in a formal training session while employed. As an illustration, informal learning can occur during meetings, customer interactions, mentoring, peer-to-peer interactions, and task exploration (Marsick and Volpe, 1999; Ellinger, 2005).

## 3. Research method

### 3.1. Qualitative research approach

Qualitative research approaches are essential for constructing theories in complex and extensive fields (Yin, 2016). Therefore, a combination of critical incident and narrative inquiry approaches (Silverman, 2011; Yin, 2015) was employed to gain insight into, and construct theories around, the cross-cultural adjustment of Chinese SIEs and OEs. We used semi-structured interviews to collect data for this research from high-management Chinese SIEs, and OEs employed in distinct Chinese companies in Pakistan. According to Selmer (2006), employing qualitative strategies may aid the researcher in overcoming “linguistic difficulties” when gathering information and assist Chinese expatriates in understanding the study issue by easing their worries. The research employs two distinct methodologies comprising “narrative analysis” that was included in the research framework (Murray, 2009; Silverman, 2011), and this strategy was utilized to encourage respondents to convey their views throughout the discussion boldly (Yin, 2015).

### 3.2. The study configurations

As stated earlier, this investigation emphasizes Chinese SIEs and OEs who have worked for various Chinese companies in Pakistan. Due to strict security standards, most of these enterprises are based in the capital of Pakistan, i.e., Islamabad, although some are located in Lahore, Karachi, and other significant areas (Sial, 2014; Arduino, 2017). The enterprises’ largest and most significant areas are involved in the infrastructure and power sectors.

Due to its efficacy in qualitative studies, the “snowballing sampling approach” was utilized to get accessibility to more responders (Glassock and Fee, 2015). To do this, the researcher interviewed all the respondents and asked whether they knew any individual who might benefit the study (Green et al., 2010). Also, research in this area (Inkson and Myers, 2003; Myers and Pringle, 2005) has demonstrated that this sampling method is efficient.

### 3.3. The participants

The following key selection parameters have been used to enrich, organize, and inform the data acquired, resulting in a thorough evaluation and explanation of the topic under investigation in this study. To begin, the first parameters for participation in the research were set as at least 1 year of employment in Pakistan as a member of the high-level management department. They relied on literature indicating that expatriates should spend at least several months in a host nation to grasp the cross-cultural atmosphere sufficiently (Firth et al., 2014). Secondly, the respondents must be able to communicate in English because all the discussions are undertaken in that language.

### 3.4. Interview process

Before conducting interviews, every participant was assured of their privacy and confidentiality and given a thorough explanation of the study’s goals. After obtaining approval from each respondent, they were given a “consent letter” to read and sign before their interviews were recorded. In addition to recording the interviews verbally, we also took notes on the respondents’ nonverbal cues, such as their posture and facial expressions, which we matched to the transcripts to ensure we captured the participants’ true feelings.

Most of the interviews took place in restaurants where the participants were most comfortable; however, some also took place inside firms. The length of the interviews ranged around 17 to 39 min. The overall length of all interviews was 964 min, with an average interview duration of approximately 27.5 min. There were 28 men and 7 women in the sample, and their average years of employment experience in Pakistan was 4.07 years. Of those interviewed, 4 had less than 2 years, 12 had between 2 and 3 years, and the remaining 19 had more than 3 years of employment experience in Pakistan. To protect the anonymity of the respondents, they were classified as either “CMx-type” (for Chinese male respondents) or “CFx-type” (for Chinese female respondents) and “type of expatriates,” while x indicates the number of respondents, respectively. Table 1 represents the participants’ demographics and provides information about the interviewees and the length of the interviews.

**Table 1:** Participants demographics.

Identity of participants	Previous experience	Time spent in the host country (Years)	Duration of interviews (minutes)	Number of words transcribed
CM1-SIE	No	3	30	2,347
CM2-SIE	No	3.5	25	1718
CM3-SIE	No	1.5	28	2073
CM4-SIE	Yes	2	35	2,432
CM5-SIE	Yes	3	37	2,741
CM6-SIE	Yes	5	34	2,406
CM7-SIE	Yes	3	39	2,724
CM8-SIE	No	2.5	25	2,146
CM9-SIE	No	6	26	2,290
CM10-SIE	Yes	5	24	1,973
CF11-SIE	No	3	28	2,396
CM12-SIE	Yes	5.5	29	2,597
CM13-SIE	No	2	30	2,752
CM14-SIE	No	8	32	2,673
CM15-SIE	No	4	37	3,091
CM16-SIE	No	3	29	2,272
CM17-SIE	Yes	10	27	2,865
CM18-OE	Yes	6	35	3,320
CM19-OE	No	4	21	1,972
CM20-OE	No	7	17	1,702
CM21-OE	No	8	28	2,229
CM22-OE	Yes	3.5	25	2,152
CF23-OE	Yes	5	19	1,890
CM24-OE	No	6	22	2,074
CF25-OE	No	1.5	26	2,218
CM26-OE	No	9	27	2,933
CM27-OE	No	4	29	3,090
CM28-OE	No	3.5	20	1,901
CF29-OE	No	2	19	1,852
CF30-OE	No	4	25	1,985
CF31-OE	Yes	2	20	2,037
CM32-OE	Yes	2	23	1,897
CM33-OE	Yes	3	36	3,075
CF34-OE	No	1	28	2,395
CM35-OE	No	1	29	2,167
Total values	Yes = 13	142.5	964	82,385
	No = 22	Average = 4.07	Average = 27.5	Average = 2353.8

### 3.5. Interview guide

To get the best answers to the study questions on the experiences of Chinese SIEs and OEs, finding the factors that would affect Chinese expatriates’ adaptability was the main goal of the interviews. The difficulties they have as they acclimate to life in Pakistan in general and their difficulties adjusting to interactions with others and their work were highlighted, along with how CCT for SIEs and OEs might assist in lessening these difficulties.

According to the results of Stage 1 of the interviews, Chinese SIEs and OEs living in Pakistan encounter various problems related to their professional and personal lives. These issues are unique to SIEs and OEs. Thus, the interview’s criteria were enhanced. Chinese expatriates were interviewed in Stage 2 to comprehend better CCT’s function in SIEs and OEs cross-cultural adjustment. The interviews aimed to gather knowledge about Chinese expatriate interactions. To encourage participants to offer “substantial insights” rather than only surface observations, questions were designed to create a comfortable environment for replying (Flick, 2009).

Therefore, the semi-structured interview questions revolved around two primary factors. The first section enquires participants’ experiences of difficulties with “general adjustment, interaction adjustment, and work adjustment elements” (e.g., what kinds of difficulties/problems were encountered). The assistance given to address these difficulties is contained in the second section, i.e., cross-cultural training (CCT). Concerning CCA, the questions focused on what the Chinese expatriates perceived as the most challenging cross-cultural practices to engage in and how successfully they transitioned, intellectually and psychosocially, to working and living abroad (Black et al., 1991). The investigators further inquired whether the company provided CCT to them or whether they self-directed their cross-cultural adjustment.

### 3.6. Data analysis procedures

This study used “MAXQDA 20” to analyze the data in this investigation, using a five-stage process (Yin, 2015). Qualitative data interpretations included 5 steps: compiling, deconstructing, reassembling, interpreting, and concluding (Rademaker, 2011). In the first step, seeking tangible outcomes for the study requires compiling the data in a usable format. Transcription was required during compiling, during which an investigator has access to the information, when information was gathered *via* interviews or focus groups (Sutton and Austin, 2015). The second phase involves deconstructing data, classifying and generating proper classifications.

Additionally, a coding procedure was used to turn the raw data into relevant information by identifying interconnected themes and concepts (Sutton and Austin, 2015). Information was reassembled in the third stage, whereby codes and categories were given significance, and the development of themes is prioritized (Braun and Clarke, 2006). Using data classified as codes and themes, the researcher must do the fourth phase, generating analytical findings (Rademaker, 2011). Finally, every study will start with a reasonable research issue in the last stage and answer it; it may only have changed significantly over the data analysis procedure (Braun and Clarke, 2012).

**Figure 1:**
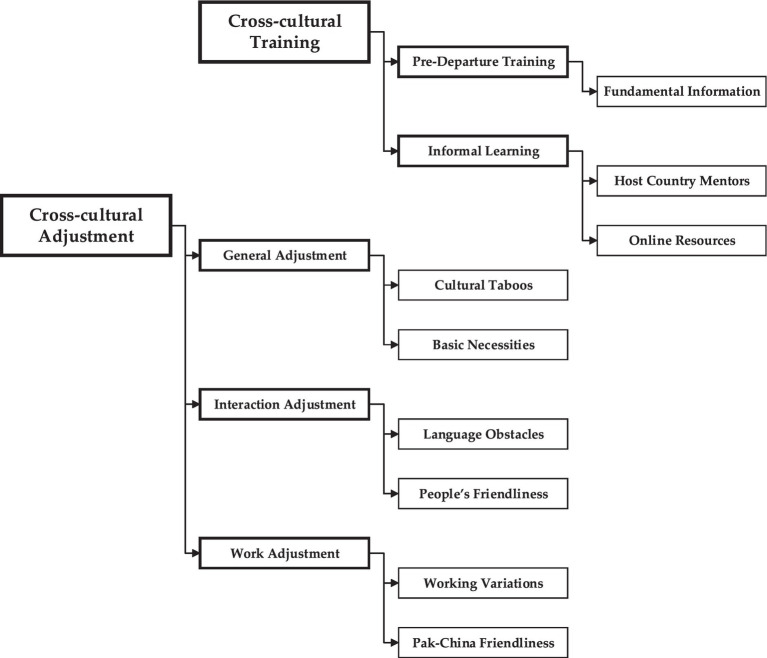
Final coding structure.

Extensive literature research was conducted before the interviews to modify the “Thematic analysis” coding scheme (King, 2004). The final form of the hierarchical coding procedure is shown in Figure 1. This portion examines the structure of multilevel coding. The analysis in each subheading summarizes the findings in each key area, and Flick (2009) “narrative arrays” display the recipient’s statements from the interviews (1).

## 4. Findings

The research findings are covered in two primary sections. The findings were outlined in the first section, which focused on the issues Chinese SIEs and OEs in Pakistan encountered throughout their CCA period, particularly those connected to their general interaction and work adjustment. The efficiency of CCT in assisting Chinese SIEs and OEs with their CCA is discussed in more detail in the second section, which also elaborates on how Chinese expatriates aid in this process (i.e., pre-departure training and informal learning).

### 4.1. Cross-cultural adjustment of SIEs and OEs in Pakistan

The CCA of SIEs and OEs varies according to their expatriation, as evidenced by their responses to queries about general adjustment, interaction adjustment, and work adjustment in Pakistan. Chinese expatriates have emphasized the following essential components are shown in Figure 1. The developed components created by the Chinese SIEs and OEs are presented in Table 2.

**Table 2:** General adjustment themes and sub-themes.

Themes	Sub-themes	Supporting quotations for each theme
General Adjustment	Cultural taboos	“Chinese people in Pakistan like to eat pork, which is prohibited, but people in this country do not consume pork due to their religious and cultural beliefs.” (**CM19-OE**)
		“When we first arrived, people advised us not to shake hands, especially with Chinese females because of their culture men could not shake hands with a female.” (**CM15-SIE**)
		“Pakistan is an Islamic state with a distinctive culture; as a result, visitors should exercise caution while interacting with locals. For instance, a friend of mine warned me that women would not appreciate it if I extended my hand to shake.” (***CM6-*SIE**)
		“It’s important to remember that being a woman in a rural area means wearing the region’s traditional garb, which may include a shalwar kameez, a dupatta, and a veil. Big cities pose no such challenges, but tiny towns may treat you differently because of your gender.” (***CF*23-OE**)
	Basic necessities	“Islamabad has a pleasant climate (neither too cool nor too warm), and the local cuisine is superb, especially karahi, naan, and BBQ, among many other options.” (***CM*4-SIE**)
		“Once I was ill, I visited the hospital, and the doctors there prescribed me some medicines and gave me some good advice; the next day, I felt much better, and the doctors in Pakistan are experts, so I was pleased with the medical care I received.” (**CM14-SIE**)
		“When first I arrived in Pakistan, the cuisine and atmosphere of Pakistan were unfamiliar to me, but after 7 years in the country. I am now accustomed to both the food and surroundings.” (**CM20-OE**)
		“Currently, Pakistan’s government is conducting control and surveillance procedures on the hygiene and food beneath the restaurants, which could improve the hygiene of Pakistani food establishments.” (**CF23-OE**)

#### 4.1.1. General adjustment elements of SIEs and OEs

The findings show that “cultural taboos” were a vital component of both SIEs and OEs’ CCA to Pakistan. For instance, Chinese expatriates were highly cautious about the do’s and don’ts when living in a multicultural environment. Whereas they have to be familiar with the culture and religion that Pakistanis follow. The majority of interviewees stated that since culture is such a delicate subject in any nation, they must be highly cautious while discussing Pakistan’s culture. Since most Pakistanis are Muslims, numerous activities are forbidden there, and more than 25 responders have noted this. Thus, the indigenous culture should be respected by them.

Before arriving in Pakistan, Chinese expatriates were concerned about issues related to food, health and atmosphere. Nevertheless, the vast number of interviewees made it apparent that they did not worry about these when they were in Pakistan. In addition, the interviewees indicated that, although what they regarded as cultural taboos, the availability of “basic necessities” assisted Chinese expatriates in Pakistan in adjusting more effectively. Islamabad, on the other hand, is a lot more reliable area to live and work in terms of security and availability of basic necessities, so that is where most of them are. Table 2 contains several examples of quotes from Chinese SIEs and OEs.

#### 4.1.2. Interaction adjustment elements of SIEs and OEs

The observations addressing the interaction adjustment of SIEs and OEs revealed that “language obstacles” are the biggest hurdles when staying in Pakistan. On some level, this element has a more significant impact on the interaction adjustment of OEs than SIEs, as SIEs are more accustomed to interacting with locals and feel more at ease than OEs. However, many responders, both SIEs and OEs, state that language obstacles are the most challenging aspect. Since Urdu is the primary spoken language of the locals, understanding it presents problems for interaction adjustment and hinders their easy integration into Pakistan.

As opposed to this, 28 interviewees emphasized that “people’s friendliness” at the workplace and outside was the main element that contributed favorably to improving both SIEs and OEs interaction adjustment. Respondents emphasized that the welcoming nature of the host culture infused their daily lives with renewed vigor. The Chinese expatriates could better integrate into the host society because of the warm welcome they received from the host people living and outside their workplace. One of the best ways to learn about a foreign lifestyle and culture is *via* direct experience and interaction with host people. Table 3 contains several examples of quotes from Chinese SIEs and OEs.

**Table 3:** Interaction adjustment themes and sub-themes.

Themes	Sub-themes	Supporting quotations for each theme
Interaction adjustment	Language obstacles	“The primary issue is that most of the population speaks Urdu, which Chinese people cannot understand.” (***CM*8-SIE**)
		“Many Pakistani locals, including several drivers, are unable to understand English and can only communicate with us by using phrases like “where,” making it difficult to go around the city.” (***CM*29-OE**)
		“The biggest issue is communication; many locals know English, but many others do not; here, Urdu is widely used, making it difficult for Chinese to understand locals.” (***CM*22-OE**)
		“I believe that learning a new language is the hardest part, as the local English is not comparable to that used in various areas of the world, and the accent also differs. However, I believe that after 3 months, all was good, and I can now speak local English with ease.” (***CF34***-**OE**)
	People’s friendliness	“I connect with my Pakistani coworkers and outside with local people, who I truly enjoy engaging. These contacts assist me in my improved transition here in Pakistan, and I acquire a great deal of knowledge from them.” (***CM12***-SIE)
		“Integrating with new people may be challenging in any culture and country, but I found it rather simple in Pakistan since everyone is so warm and welcoming to my fellow Chinese and me.” (***CM31***-OE)
		“We communicate with our coworkers, and they are all delightful. However, when we go out for lunch or dinner, we also engage with locals, who are all quite kind and welcoming.” (***CF***34-OE)
		“When I first arrived in Pakistan’s Gilgit area, I could not communicate in English, but locals and individuals from their circles of influence gradually taught me and provided more assistance.” (***CM17***-SIE)

#### 4.1.3. Work adjustment elements of SIEs and OEs

The study’s results on SIEs and OEs’ work adjustment revealed that “work variations” had a detrimental effect on people’s ability to adapt to Pakistan’s working environment. These challenges will arise due to the wide variations in cultural customs, beliefs, and morals that characterize the workplaces of people from various backgrounds and cultures. The vast majority of respondents emphasized worries about work variation. Over 20 responders mentioned this difficulty, citing the disparities in business practices and cultural norms are the main reasons. The disparity in working hours between the two nations can be one of the causes. Most interviewees concur that Pakistani professionals prioritize creating social networks, arriving late to their workplaces, and delaying their duties to complete them on time.

However, “Pak-China friendliness” has been cited as a positive factor by both SIEs and OEs and is significantly influencing the workers’ ability to acclimate to life in Pakistan. Additionally, they were passionate in their comments on the friendship between Pakistan and China. Most of them believed that they were in charge of preserving and enhancing this relationship, and they had to tread cautiously in Pakistan to avoid behaving in a way that would harm relations between Pakistan and China. The mutually beneficial relationship between China and Pakistan has facilitated the integration of Chinese SIEs and OEs into Pakistani society and encouraged more exposure to Pakistani culture. Table 4 contains several examples of quotes from Chinese SIEs and OEs.

**Table 4:** Work adjustment themes and sub-themes.

Themes	Sub-themes	Supporting quotations for each theme
Work adjustment	Working variations	“The biggest hurdle is keeping the same work hours as Pakistanis in China. When they leave, we keep working, but we cannot concentrate yet we are alone. This is the obstacle in the workplace, in my opinion.” (***CM1*2-SIE**)
		“The local employees here do not work effectively, or rather, they work slowly; thus, for me, this is an issue in the workplace.” (***CM*16-SIE**)
		“Pakistani people do not intervene with your job if you are in the financial department, but Chinese people work as a group and are very hard-working. I do not want to imply that Pakistani people aren’t, but they are relaxed and handle their work differently. So this is the notion of work variation.” (***CF***30-OE)
		“Time management is an issue here; people frequently arrive late for appointments, and when they do show up for meetings, they sometimes claim to be unavailable.” (***CM*28-OE**)
	Pak-China Friendliness	“We are great neighbors!! We love one another!! People of Pakistan are very friendly to Chinese people; when they meet with us, they are desirous of taking group photos with us. They offer a cup of tea to us. This is an index of their sincerity with us.” (***CM*32-OE**)
		“Pak-China relations are above trades and profits. We are believers and practitioners to do for the best of Pakistan. We want to see a healthier, happier, more prosperous neighboring country. The stronger Pakistan is a matter of immense pleasure for the people and the Republic of China.” (**CM24-OE**)
		“I am convinced that Pakistanis are genuine and nice people. Without question, Pakistan is a civilized and economically evolved country.” (***CM5***-**SIE**)
		“Yeah! We love and respect Pakistan. Brethren country for Chinese. We are good friends just like brothers.” (***CM*35-OE**)

### 4.2. Cross-cultural training elements

CCT is a set of formally determining actions to educate workers for more genuine social interactions and employment success when they often interact with colleagues from other cultures who work in diverse environments. The world is becoming more globalized; people live and work in different cultural surroundings from their native countries. Thus, CCT seeks to instill confidence in expatriates in their international tasks. Furthermore, this training will enhance their adaptability, understanding, and awareness from many perspectives. The observations are reported in two sections (i.e., pre-departure training and informal learning of SIEs and OEs).

#### 4.2.1. Pre-departure training

Results from pre-departure training shed light on strategies employed by foreign nationals to succeed in their new environment (i.e., basic information). Typically, the company or a training institution provides pre-departure training. This is done to aid in successfully adjusting to working and residing abroad and, most significantly, the host nation’s culture. According to the study’s outcomes, most Chinese OEs were trained before being assigned to the foreign mission. The respondents who led the pre-departure training expressed that the background knowledge about the host nation assisted them in adjusting to the employment and residential situations and offered suggestions on how to interact or conduct with them. Table 5 contains several examples of quotes from Chinese SIEs and OEs.

**Table 5:** Cross-cultural training themes and sub-themes.

Themes	Sub-themes	Supporting quotations for each theme
Pre-departure training	Fundamental information	“I came from a huge company, so there was a complete training package before we arrived here. In this training, different sessions were delivered to us, which taught us to respect the host culture and respect people over there.” (***CM1*9-OE**)
		“Whenever we travel to any foreign nation, it is a form of training. Usually, the firm in China would handle this for us, and the main crucial aspect of the training is specifically to embrace the culture; even if you do not know it, you must accept it, so I believe that’s helpful.” (***CM*30-OE**)
		“We undergo one week of instruction in China because China and Pakistan have distinct cultural traditions because most individuals are Muslims in Pakistan. We must know more about Muslim and Pakistani culture as we have not previously interacted with Muslim friends and families.” (***CM*26-OE**)
Informal learning	Host country mentors	“To better understand Pakistani culture and way of life, I have relied on what my coworkers have taught me about Pakistani history, religion, customs, and festivals since being here.” (***CM2*4-OE**)
		“I learn much about other people’s religions and customs from my friends, and my coworkers always have interesting discussions about these topics during tea break.” (***CM*13-SIE**)
		“As you are aware, the Pakistani business is unique; thus, a few of our workers have much knowledge; they offer us recommendations and advise on how to operate the Pakistani business, which aids us in adjusting to Pakistan.” (***CM*3-SIE**)
		“When I came here, the local distributor assigned two individuals to assist me. I learned a lot from them, especially about the way of life and the culture. I also attended several Pakistani weddings and am fond of Pakistani wedding culture.” (***CF*25-OE**)
	Online resources	“I did not go through any cross-cultural training, but I’ve gained so much knowledge about Pakistan thanks to the internet, which is the sole reliable source of information about the country.” (***CM16***-**SIE**)
		“I did not attend such orientation or cross-cultural training before traveling to Pakistan; instead, I conducted my online research.” (***CM*1-SIE**)
		“I am familiar with Pakistan and the locals, and before arriving, I conducted my independent online research and did not get any training.” (***C*M6-SIE**)
		“To learn about Pakistani culture while moving here, I did some online research and engaged in several social media sites. This has been quite helpful.” (**CM10-SIE**)

#### 4.2.2. Informal learning

Informal learning is becoming increasingly significant in today’s technologically advanced, globally interconnected environment, where people may acquire it in various ways. According to the findings, “host country mentors” and “online resources” are effective forms of informal learning. HCNs familiar with the host country’s lifestyle and culture, also known as the “host country mentor” can aid expatriates in adjusting. A majority of participants said that for expatriates, interactions with HCNs in the workplace and in daily life are an unavoidable part of their missions abroad. These interactions provide us with knowledge about their working and non-working atmosphere. There are several methods employed for informal learning. Most interviewees thought that they used the internet as a platform to know more about Pakistan and its culture. Yet, in the modern world, internet technology is a popular method that most individuals utilize to stay informed about what is going on around the globe. Table 5 contains several examples of quotes from Chinese SIEs and OEs.

## 5. Discussion

This study looked at their distinctions to understand how SIEs and OEs adjust to Pakistan’s culture. Furthermore, the study is concerned with determining the amount of CCT that will facilitate Chinese SIEs and OEs.

This approach is validated by the literature and contributes to it by developing a step-by-step method for comprehending the CCA of Chinese SIEs and OEs. Literature indicates that expatriates experience a variety of obstacles during their transition to the host country (Zhang and Guttormsen, 2016; Nadeem and Mumtaz, 2018; Noman et al., 2020). The most challenging adjustment issues for Chinese expatriates in Pakistan were cultural taboos referring to general adjustment and language obstacles in interaction adjustment. Finally, work variations are linked with work adjustment. Despite this, they managed to adapt effectively due to various circumstances, such as the availability of basic necessities, the peoples’ friendliness and the friendly relations between Pakistan and China, which benefited both Chinese SIEs and OEs in Pakistan.

By examining the causes of variations in expatriate experiences across OEs and SIEs, this study goes above direct correlations and significantly adds to the body of knowledge. Even though OEs and SIEs have significant disparities from one another (Peltokorpi and Jintae Froese, 2009; Biemann and Andresen, 2010; Doherty et al., 2011), prior studies have not clarified why these variations in expatriate experiences exist between SIEs and OEs. According to the current research, SIEs have stronger interaction adjustment because of higher host language ability and a brotherly Pak-China relationship. The findings suggest that SIEs are well acclimated to interaction and general adjustment than OEs, maybe because they frequently interact with locals and stay in the local area. Peltokorpi (2008) asserts that working in a local setting gives SIEs opportunities to engage with locals, allowing them to pick up and adapt corresponding interactional habits. Conversely, OEs could view their overseas posting as a crucial phase in their employment and their interactions with Pakistanis outside of work less significant. OEs may be more likely to contact other expatriates throughout their predetermined duration of expatriation in Pakistan.

Like other studies (Suutari and Brewster, 2000; Jokinen et al., 2008), the results reveal that work characteristics vary. In other words, OEs usually hold senior roles and operate in overseas companies more often. This may partially be due to the frequent dispatch of OEs to manage and transmit information to overseas companies (Edström and Galbraith, 1977). Our results support earlier research (Peltokorpi and Jintae Froese, 2009; Biemann and Andresen, 2010; Froese and Peltokorpi, 2011), which found that SIEs exhibit more significant levels of interaction and general adjustment but lower overwhelmingly favorable work sentiments, particularly in terms of work happiness, than OEs.

The second objective of this study is to improve the CCA of Chinese SIEs and OEs in the host nation. The literature indicates that CCT is a valuable strategy for supporting expatriates’ CCA. Additionally, our research demonstrates that CCT makes expatriates seem more relevant while working abroad (Caligiuri et al., 2005; Noman et al., 2020). This research focuses on the two approaches that expatriates learn about and get familiar with the host nation (i.e., pre-departure training and informal learning). Firms or professional institutions often give pre-departure training; thus, we deduce that this mode of learning is formal, which firms deliver before the departure of expatriates (Tissot, 2008). The findings reveal that Chinese expatriates who received pre-departure training settled well in Pakistan (see Table 5). Previous research has also proven that this first cross-cultural training gives fundamental knowledge of the host nation, which is required for expatriates’ initial training on arriving at their planned destination. For example, these training strategies may assist expatriates in learning about cultural beliefs and norms, the country’s corporate procedures, dressing conventions, vital rituals, and primary language understanding (Avril and Magnini, 2007).

However, the study also emphasizes how expatriates acquire informally. These informal teaching strategies examined in this study are typically organized by expatriates individually. The findings on informal learning revealed that Chinese expatriates adopt informal learning methods both before and after their entrance to Pakistan (i.e., host country mentors and online resources). The results suggest that the guidance offered by host country mentors assists expatriates in adjusting to their new environment. Moreover, expatriates might benefit more from mentor support networks in their host nation. The findings show that expatriates receive information from their host country mentors about their way of life, culture, and business practices for their simple integration into the host nation. Furthermore, Bear (2008) study shows that in the digital era, most expatriates nowadays acquire crucial information about their host location through informal learning utilizing online technology (i.e., Google, Youtube, Facebook, Whatsapp, Webchat, e-mail, and blogs).

## 6. Conclusion, practical implications, and limitations of the study

### 6.1. Conclusion

The current study centered on the qualitative data analysis of 35 Chinese expatriates in Pakistan, with the sample consisting of 17 SIEs and 18 OEs. To build a thorough knowledge of the CCA process of expatriates, the variations between SIEs and OEs’ CCA, and the moderating impact of CCT in helping expatriates in CCA. This research adds to our knowledge and comprehension of the variations in CCA between SIEs and OEs. Despite the demographics and cultural backgrounds of SIEs and OEs being quite similar, SIEs have more excellent local knowledge, and OEs operate in a more organizational atmosphere. We contributed to the literature by explaining why expatriate results vary between SIEs and OEs. The results imply that SIEs have better general and interaction adjustment than OEs. In addition, OEs are more successful in work adaptation. However, Chinese companies are increasingly expanding their operations outside of Asia and Africa. Therefore, we recommend that companies create various CCT programs depending on the various sorts of expatriates before and after arriving in the host country. We anticipate that CCT will benefit expatriation as well as the overall organization. The CCT will ease the adjustment process of expatriates and give them a basic grasp of the culture of their new home. Language proficiency is also necessary for expatriates to understand the culture of their new home. To ensure the performance of their foreign mission, companies may provide language training and engage both SIEs and OEs, regardless of the location of the training.

### 6.2. Practical implications

There are several practical implications in this study. First, the ability to think and act in ways that are appropriate in other cultures is essential for thriving in a new environment. Expatriate employees would benefit more from their overseas missions if they received language and cultural training prior to leaving for the host country. This is because such training helps participant’s better grasp the customs and norms of the host culture and its inhabitants (Caligiuri et al., 2001; Selmer, 2009; Selmer and Lauring, 2015; Liu et al., 2018). Second, interpersonal interactions involving expatriates and host peoples enable expatriates to strengthen their bonds with them; as a result, hosting firms must propose numerous efforts to boost interaction between expatriates and HCNs. The effort might include teaching local workers some basic Chinese and the Chinese workers some basic Urdu. This will assist in reducing the interaction gap between local and Chinese workers. Third, firms should engage host peoples to facilitate the expatriates’ social transition *via* promotinging social ties. For instance, by bringing close family members to organizational activities, Chinese expatriates and local employees might establish a feeling of a friendly atmosphere. Finally, this will aid in developing cultural awareness and reducing adjustment issues in the host environment.

### 6.3. Limitations and future research directions

This section elaborates on this study’s limitations and future research directions. First, our study employs qualitative methodologies, with data gathered through semi-structured interviews with top-level Chinese SIEs and OEs operating in Pakistan. We concentrated on Chinese workers who speak English, making it easier to understand that their difficulties and adaptation differed depending on their encounters. Future researchers may investigate non-English speaking Chinese expatriates, e.g., by analyzing their adaptation process and identifying their obstacles with those of English-speaking Chinese expatriates. Our focus on top-level expatriates aided in the development of an extensive understanding of their adjustment; however, the adjustment of expatriates working at various levels and in different roles varies. As a result, future research will perform an investigation that takes into account many levels, such as defining the process of adaptation and acknowledging that the problems of adaptation vary depending on the management levels.

Second, the research methodology centered on Chinese expatriates working in Pakistan in managerial positions and encouraged participants to express their own experiences and thoughts. Due to their heavy workload, time constraints, and security issues, some respondents could not share important information regarding their encounters. This shortcoming was addressed by conducting more interviews and analyzing unstructured memoranda in the mix of collected data. Therefore, it is suggested that future studies incorporate expatriates and their coworkers in a research design to verify results with another person the respondents dealt with daily. Additionally, if access can be gained, combining several qualitative methodologies, such as observations and interviews, may help investigators better understand the causes and the adaptation mechanism.

Third, the Chinese expatriates who participated in this study came from various regions of China. Most came from big cities, while others were from small areas. In addition, some resided nearer to the Pakistan-China border. These distinctions may have had a distinct effect on respondents’ exposure and information level. Although this study did include several features, location-based impacts were not analyzed since no information was gathered regarding the city in which each expatriate resides. It is advised that future studies consider those location-based variances to further their comprehension of how expatriates adapt to their new environment. Fourth, almost all other studies of expatriates, including this one, only gathered data once. In order to offer more convincing evidence of correlation and to effectively comprehend the temporally dependent procedure of CCA, longitudinal data would be beneficial. Finally, research has shown a strong correlation between CCT and expatriates’ adjustment, even though there are limited Chinese SIEs and OEs. Therefore, quantitative approaches may use in future studies to examine the findings of this research.

## Data availability statement

The original contributions presented in the study are included in the article/supplementary material, further inquiries can be directed to the corresponding author.

## Ethics statement

Ethical review and approval was not required for the study on human participants in accordance with the local legislation and institutional requirements. The patients/participants provided their written informed consent to participate in this study.

## Author contributions

All authors listed have made a substantial, direct, and intellectual contribution to the work and approved it for publication.

## Funding

This work was funded by Princess Nourah bint Abdulrahman University Researchers Supporting Project number (PNURSP2022R4), Princess Nourah bint Abdulrahman University, Riyadh, Saudi Arabia.

## Conflict of interest

The authors declare that the research was conducted in the absence of any commercial or financial relationships that could be construed as a potential conflict of interest.

## Publisher’s note

All claims expressed in this article are solely those of the authors and do not necessarily represent those of their affiliated organizations, or those of the publisher, the editors and the reviewers. Any product that may be evaluated in this article, or claim that may be made by its manufacturer, is not guaranteed or endorsed by the publisher.
